# Prognostic Value of Phosphatidylinositol-3 Kinase p110 α Protein Expression in Patients with Stage I–III Invasive Breast Cancer

**DOI:** 10.3390/cancers18020301

**Published:** 2026-01-19

**Authors:** Zhiqiang Zong, Xuan Zhou, Jian Shen, Min Yan, Xi Xia, Jingjing Li, Xian Wang, Fanfan Li

**Affiliations:** 1 Department of Oncology, The Second Affiliated Hospital of Anhui Medical University, No. 678 Furong Road, Hefei 230601, China; 2 Department of Pathology, The Second Affiliated Hospital of Anhui Medical University, No. 678 Furong Road, Hefei 230601, China

**Keywords:** p110α, breast cancer, overall survival, recurrence-free survival, prognosis, immunohistochemistry

## Abstract

Background: The prognostic value of phosphatidylinositol-3-kinase p110α, a key catalytic subunit in the PI3K/AKT pathway, in breast cancer remains controversial. This study evaluated its prognostic significance in stage I–III invasive breast cancer. Methods: p110α protein expression was detected via immunohistochemistry (IHC) in 161 patient tissue samples. Its association with overall survival (OS) and relapse-free survival (RFS) was analyzed using Kaplan–Meier and Cox proportional hazards models. Results: p110α positivity was detected in 59.0% of specimens and showed significant correlation with histological grade (*p* = 0.034). Survival analysis revealed that p110α positivity was associated with worse OS (log-rank *p* = 0.008) and RFS (log-rank *p* = 0.018). In multivariate analysis, p110α expression was an independent predictor of poor prognosis for both OS (HR = 2.45, 95%CI: 1.25–4.78) and RFS (HR = 2.12, 95%CI: 1.14–3.94). This association with poor prognosis was particularly pronounced in stage I–II, hormone receptor (HR)-positive, and human epidermal growth factor receptor 2 (HER2)-negative subgroups. Supporting evidence from the PROGgeneV2 database showed that high *PIK3CA* mRNA levels predicted inferior survival in external cohorts. Conclusions: p110α protein expression is an independent biomarker for adverse outcomes in stage I–III invasive breast cancer. Its assessment could improve prognostic evaluation and guide personalized therapy.

## Simple Summary

Breast cancer outcomes vary widely, and better predictors are needed to guide treatment decisions. p110α is a key protein involved in cell growth signals, often altered in cancer. This study investigated whether the expression levels of p110α protein in tumors could help predict the prognosis of patients with early-stage breast cancer. We analyzed tissue samples from 161 patients and found that higher p110α levels were associated with shorter survival and higher risk of recurrence. This finding was confirmed using separate public data. Measuring p110α could therefore help doctors identify patients with a more aggressive disease, potentially leading to more personalized and effective treatment strategies.

## 1. Introduction

Globally, breast cancer maintains its status as the most commonly diagnosed cancer and a predominant cause of cancer-associated deaths in the female population [1,2,3]. While diagnostic techniques and comprehensive treatment modalities have evolved remarkably, the heterogeneity of the disease and its distinct molecular subtypes continue to pose significant challenges to predicting patient outcomes [4]. Hence, identifying reliable biomarkers is crucial for enhancing prognostic accuracy and guiding personalized treatment strategies.

At the molecular level, dysregulation of the phosphoinositide 3-kinase (PI3K)/AKT pathway, which orchestrates cell growth, survival, and metabolism, is a well-established driver of cancer pathogenesis across a wide spectrum of human cancers, including breast cancer [5,6,7,8]. In breast cancer specifically, it represents one of the most frequently altered signaling pathways, playing a pivotal role in tumor initiation, progression, and therapeutic resistance [5]. The p110α catalytic subunit, encoded by the *PIK3CA* gene, is a critical factor in this pathway [9]. *PIK3CA* ranks among the most commonly mutated oncogenes in human cancers, and in breast cancer, its frequent activation via gain-of-function mutations or overexpression constitutes a major oncogenic driver [10,11]. This activation promotes tumor progression and has been implicated in resistance to conventional therapies [12]. While the prognostic and predictive implications of *PIK3CA* mutations have been extensively studied [13,14], the clinical significance of the encoded p110α protein, which reflects the functional effector of pathway activation, has received considerably less attention, and its independent prognostic value in early breast cancer is poorly defined. Thus, a systematic evaluation of p110α protein expression is warranted to clarify its role.

The primary objective of this study was to elucidate the prognostic role of p110α protein expression in a well-characterized cohort of patients with stage I–III invasive breast cancer (IBC). We systematically detected p110α levels by immunohistochemistry (IHC) and analyzed their association with clinicopathological parameters and survival outcomes. To strengthen the biological plausibility and generalizability of our findings, we complemented our protein-level analysis with external validation at the transcriptional level, examining the association of *PIK3CA* mRNA expression with survival outcomes in large, independent public cohorts. Our objective was to establish p110α as a clinically applicable biomarker for improved prognostic assessment in early IBC.

## 2. Methods

### 2.1. Study Population

This single center, retrospective study was conducted on a cohort of female breast cancer patients who received surgical resection at the Second Affiliated Hospital of Anhui Medical University between January 2017 and December 2023. A total of 161 female patients were included in the final cohort. Their ages ranged from 26 to 77 years, with a median age of 50 years. All patients had pathologically confirmed stage I–III IBC. To be eligible, patients must meet the following criteria: (1) pathologically confirmed invasive breast cancer; (2) classified as stage I–III based on the AJCC (8th edition) staging system; (3) receipt of definitive breast cancer surgery (e.g., lumpectomy, mastectomy, or modified radical mastectomy); (4) completion of standard adjuvant therapy as clinically indicated. Exclusion criteria were as follows: (1) incomplete clinicopathological data or follow-up information; (2) a concurrent or prior history of another malignant tumor.

### 2.2. Follow-Up

Patients were followed until September 1st, 2025. The main outcome measures were overall survival (OS) and recurrence-free survival (RFS). OS referred to the duration from surgery to death from any cause. RFS was defined as the interval from surgery to the initial occurrence of either disease relapse (local, regional, or distant) or death from any cause.

### 2.3. Immunohistochemistry (IHC)

Archival Formalin-fixed paraffin-embedded (FFPE) breast cancer tissue blocks were retrieved from the Department of Pathology. IHC staining for p110α protein was performed on 4-μm tissue sections according to standard protocols. Briefly, after deparaffinization, rehydration, and antigen retrieval, sections were incubated with a primary antibody against p110α (Anti-*PIK3CA*/p110α antibody [GB11769] [15], dilution 1:300, Wuhan Servicebio Technology Co., Ltd., Wuhan, China). Staining was then visualized using an appropriate detection system (DAB chromogen).

Two independent, blinded pathologists assessed p110α immunoreactivity without prior knowledge of patient outcomes. In case of discrepant scores, a third pathologist was consulted to reach a consensus. The staining results were assessed under high-power fields (400× magnification), with five non-overlapping fields randomly selected per case. A semi-quantitative scoring system was used, considering both staining intensity and the percentage of positive tumor cells. First, staining intensity was graded on a scale of 0 to 3: 0 (no staining), 1 (weak, light brown), 2 (moderate, brown), and 3 (strong, dark brown). Second, the percentage of tumor cells exhibiting any positive staining was scored on a scale of 0 to 4: 0 (0%), 1 (1–25%), 2 (26–50%), 3 (51–75%), and 4 (76–100%). The final IHC score for each case was calculated by multiplying the intensity score by the percentage score, yielding a possible range of 0 to 12. Based on established cut-offs from prior studies and our internal validation, a final score of < 4 was classified as negative for p110α expression, and a score of ≥4 was classified as positive.

### 2.4. External Validation Using Gene Expression Data

To independently validate the prognostic significance of our findings at the transcriptional level, we utilized the open access gene expression portal PROGgeneV2 (https://proggene.ccbb.indianapolis.iu.edu/index.php, accessed on 3 January 2026). PROGgeneV2 is a curated online database that compiles gene expression and survival data from multiple public repositories, including The Cancer Genome Atlas (TCGA) and Gene Expression Omnibus (GEO) [16]. We queried the database for the *PIK3CA* gene using the following independent breast cancer cohorts for validation: The Cancer Genome Atlas Breast Invasive Carcinoma (TCGA-BRCA) cohort (*n*N = 594), the GSE25055 cohort (*n* = 309), and the GSE10886 cohort (*n* = 156). The hazard ratios (HRs), 95% confidence intervals (CIs), and associated *p*-values generated by the portal’s Cox proportional hazards model were recorded. This followed an assessment of the relationship between *PIK3CA* mRNA expression and both overall and recurrence-free survival in available breast cancer cohorts. This analysis served as an external, mRNA-level validation of the prognostic value identified by our protein-level immunohistochemical study.

### 2.5. Statistical Analysis

The statistical analyses were conducted with the software packages SPSS (version 26.0) and R (version 4.2.1). For variable description, continuous variables that followed a normal distribution were expressed as the mean ± standard deviation, while categorical variables were reported as numbers and percentages. Associations linking p110α expression to clinicopathological characteristics were evaluated by the Chi-square or Fisher’s exact test, based on applicability. For survival outcomes, curves were constructed using the Kaplan–Meier method, and the log-rank test compared differences between groups. Our analytical approach involved first employing univariate Cox proportional hazards regression to identify variables associated with to OS and RFS. Subsequently, any variable achieving a *p*-value under 0.05 in the univariate analysis was advanced into a multivariate Cox regression model. This final multivariate Cox model was used to identify independent prognostic factors. A two-sided *p*-value < 0.05 was considered to indicate statistical significance.

This study was conducted and reported in accordance with the REMARK (Reporting Recommendations for Tumor Marker Prognostic Studies) guidelines [17]. The completed REMARK checklist is provided as Table S1.

## 3. Results

### 3.1. Patient Characteristics

**Figure 1:**
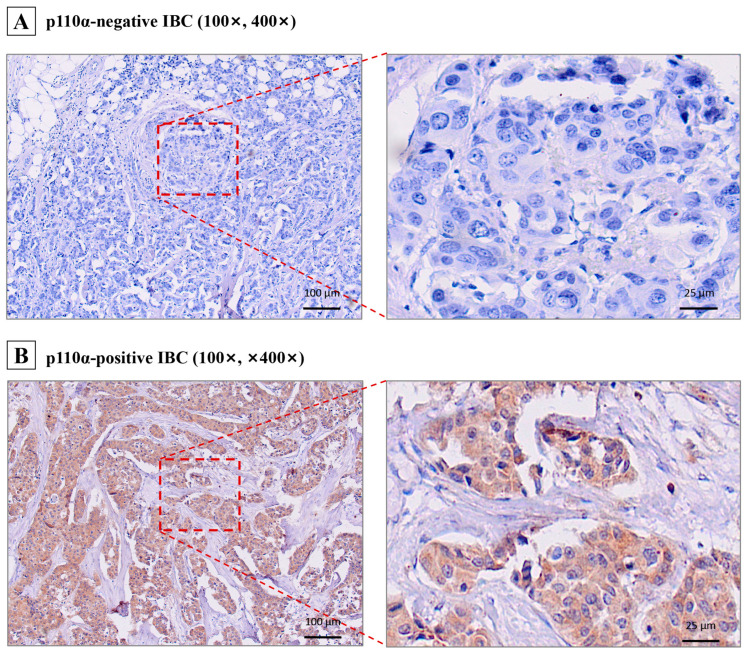
Representative IHC staining of p110α in IBC tissues. (**A**) IHC staining showed IBC cases with negative expression of p110α. The left image (100×) shows the low magnification field of view, and the box area represents the high magnification area; the right image is a high-magnification image of the corresponding area (400×). (**B**) IBC cases with positive expression of p110α, displayed in the same manner in (**A**). Abbreviations: IHC, immunohistochemistry; IBC, invasive breast cancer.

The study cohort included 161 female patients with a confirmed diagnosis of invasive breast cancer. Their ages ranged from 26 to 77 years, with a median of 50 years. At a median follow-up of 80.4 months, disease progression and death were recorded in 61 (37.9%) and 49 (30.4%) patients, respectively, as of the final follow-up date of 1 September 2025. The median OS and median RFS had not yet been reached. Immunohistochemical analysis revealed p110α positivity in 59.0% (95/161) of the invasive breast cancer tissue samples (Table 1), with representative staining images shown in Figure 1. The associations between p110α expression status (negative vs. positive) and all collected clinicopathological parameters are detailed in Table 2. p110α expression correlated significantly only with higher histological grade (*p* = 0.034) but showed no significant association with age, menstrual status, tumor size, lymph node metastasis, clinical stage, hormone receptor (HR) status, or human epidermal growth factor receptor 2 (HER2) status (all *p* > 0.05).

### 3.2. Association Between p110α Protein Expression and Patient Prognosis

**Figure 2:**
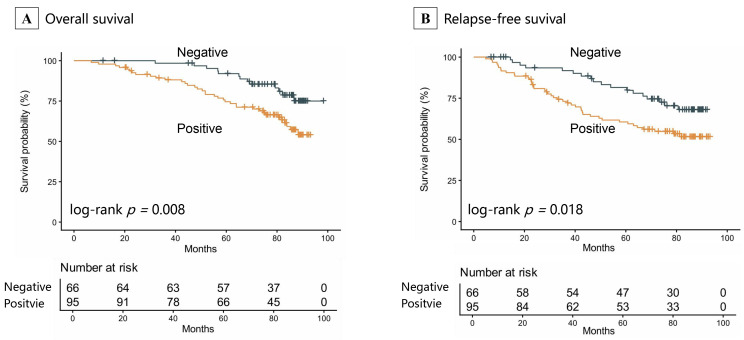
Kaplan–Meier Survival analysis for prognosis by p110α expression status. (**A**) overall survival; (**B**) relapse-free survival.

Survival analyses, utilizing Kaplan–Meier curves and log-rank tests, established a significant correlation between p110α-positive expression and unfavorable clinical outcomes, including decreased survival and increased risk of relapse (Figure 2A,B). Specifically, the p110α-positive cohort had a mean OS of 75.97 months (95%CI: 70.8–81.1), substantially shorter than the 90.68 months (95%CI: 86.8–94.6) in the negative cohort (log-rank *p* = 0.008). Correspondingly, patients with p110α-positive tumors had a significantly shortened mean RFS of 48.0 months (95%CI: 38.2–54.0), compared with 60.5 months (95%CI: 56.6–72.5) in the p110α-negative group (log-rank *p* = 0.018).

### 3.3. Univariate and Multivariate Cox Regression Analyses

Univariate analysis identified positive p110α expression (HR = 2.30, 95%CI: 1.22–4.33, *p* = 0.010), age ≥ 55 years (HR = 2.13, 95%CI: 1.13–4.01, *p* = 0.020), and histological grade 3 (HR = 2.63, 95%CI: 1.50–4.62, *p* = 0.001) to be significantly associated with worse OS. Following adjustment for significant univariate predictors, both positive p110α expression (HR = 2.00, 95%CI: 1.05–3.80, *p* = 0.034) and histological grade 3 (HR = 2.24, 95% CI: 1.26–3.97, *p* = 0.006) remained independent prognostic factors for OS (Table 3).

Regarding RFS, univariate analysis identified positive p110α expression, age ≥55 years, premenopausal status, and histological grade 3 as significant risk factors. Multivariate Cox regression identified positive p110α expression and age ≥ 55 years as independent predictors of reduced RFS, with respective HRs of 1.92 (95%CI: 1.10–3.34, *p* = 0.022) and 1.79 (95%CI: 1.07–2.99, *p* = 0.028) (Table 4).

### 3.4. Subgroup Analyses of p110α Expression

**Figure 3:**
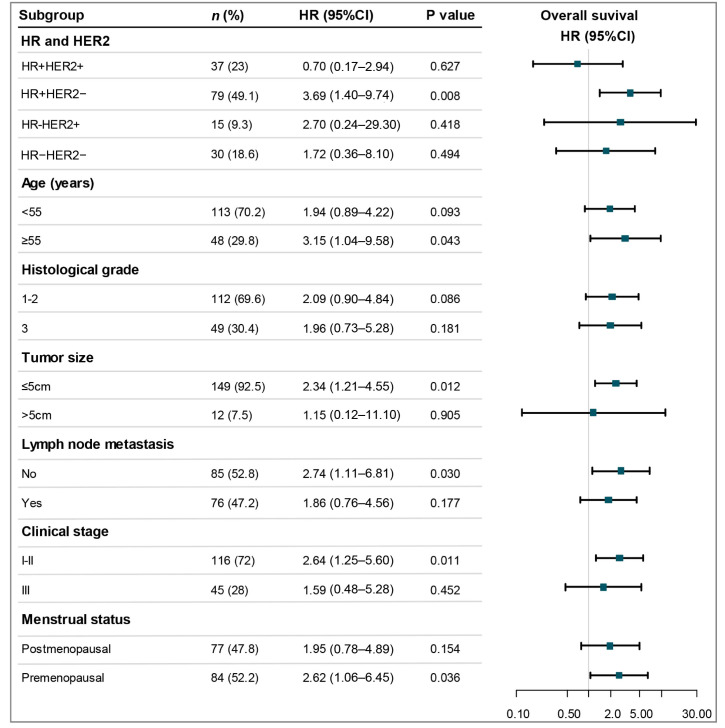
Forest plot of the association between p110α positive expression and OS across patient subgroups. An HR > 1 indicates an increased risk of mortality associated with p110α positivity. The size of the data markers corresponds to the subgroup sample size. Abbreviations: OS, overall survival; *n*, number of patients; HR (in Cox models), hazard ratio; CI, confidence interval.

**Figure 4:**
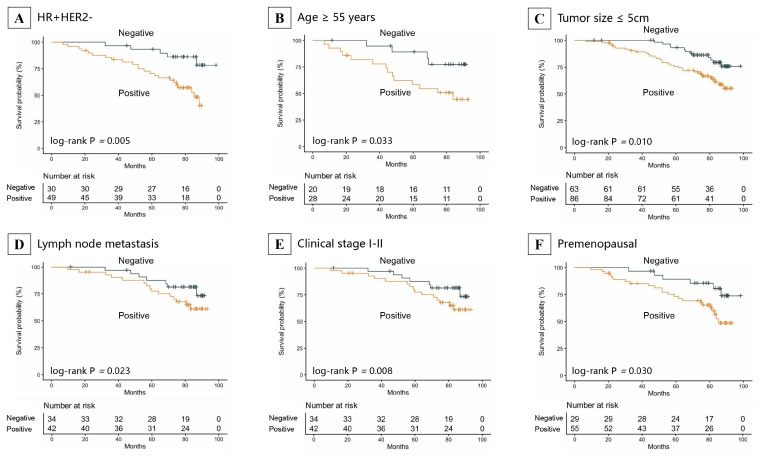
Kaplan–Meier survival analysis of OS across patient subgroups stratified by p110α expression. (**A**) HR-positive/HER2-negative subgroup; (**B**) age ≤ 55 years subgroup; (**C**); tumor size ≤ 5 cm subgroup; (**D**) lymph node metastasis subgroup; (**E**) clinical stage I–II subgroup; (**F**) premenopausal subgroup. Abbreviations: OS, overall survival; HR, hormone receptor; HER2, human epidermal growth factor receptor 2.

Subgroup analyses for OS demonstrated that p110α positivity conferred a higher risk of mortality across multiple key patient subsets (Figure 3, Table S2). Significant associations with reduced OS were observed in individuals with HR-positive/HER2-negative disease (HR = 3.69, 95%CI: 1.40–9.73, *p* = 0.008), those aged ≥55 years (HR = 3.15, 95%CI: 1.04–9.58, *p* = 0.043), premenopausal patients (HR = 2.62, 95% CI: 1.06–6.45, *p* = 0.036), as well as in those with tumor size ≤ 5 cm (HR = 2.34, 95% CI: 1.21–4.55, *p* = 0.012), lymph node metastasis negative (HR = 2.74, 95% CI: 1.11–6.81, *p* = 0.030), and stage I–II disease (HR = 2.64, 95%CI: 1.25–5.60, *p* = 0.011). The Kaplan–Meier curves in Figure 4 visually depict the consistent association between p110α positivity and poorer OS within these specific subgroups.

**Figure 5:**
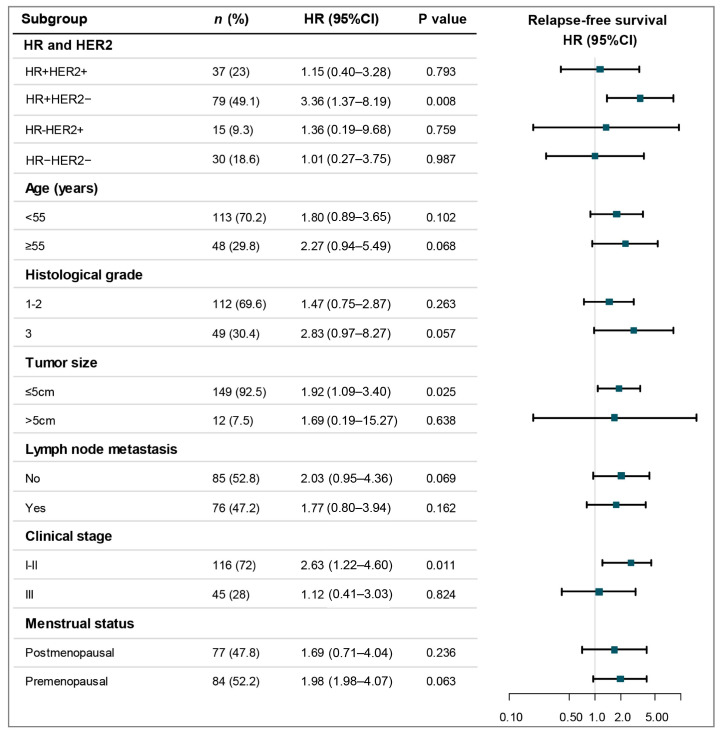
Forest plot of the association between p110α positive expression and RFS across patient subgroups. An HR > 1 indicates an increased risk of mortality associated with p110α positivity. The size of the data markers corresponds to the subgroup sample size. Abbreviations: RFS, relapse-free survival; *n*, number of patients; HR (in Cox models), hazard ratio; CI, confidence interval.

**Figure 6:**
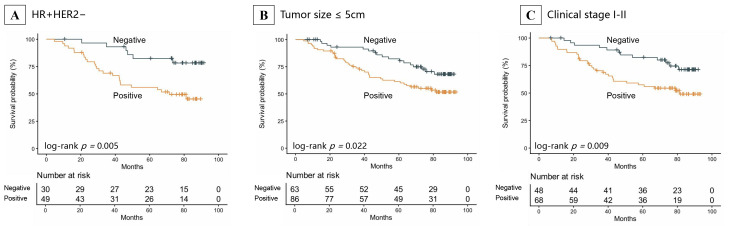
Kaplan–Meier survival analysis of RFS across patient subgroups stratified by p110α expression. (**A**) HR-positive/HER2-negative subgroup; (**B**) tumor size ≤ 5 cm subgroup; (**C**) clinical stage I–II subgroup. Abbreviations: RFS, relapse-free survival; HR, hormone receptor; HER2, human epidermal growth factor receptor 2.

For RFS, positive p110α expression predicted significantly worse outcomes in subgroups of patients with HR-positive/HER2-negative disease (HR = 3.36, 95%CI: 1.37–8.19, *p* = 0.008), tumor size ≤ 5 cm (HR = 1.92, 95%CI: 1.09–3.40, *p* = 0.025), and stage I–II disease (HR = 2.63, 95%CI: 1.22–4.60, *p* = 0.011) (Figure 5, Table S3). The Kaplan–Meier curves illustrating the adverse impact of p110α expression on RFS within these specific subgroups are depicted in Figure 6.

### 3.5. External Validation at the Transcriptional Level

To validate our protein-based findings at the transcriptional level, we analyzed the PROGgeneV2 database. Mining this resource demonstrated that high mRNA levels of *PIK3CA*, the gene encoding p110α, were significantly linked to poorer survival in independent breast cancer cohorts. Specifically, in the TCGA cohort, elevated *PIK3CA* expression was correlated with shorter overall survival (HR = 1.82, 95%CI: 1.30–2.53, *p* < 0.001; Figure S1A). Similarly, it predicted reduced recurrence-free survival in both the GSE25055 (HR = 1.62, 95%CI: 1.12–2.35, *p* = 0.011; Figure S1B) and GSE10886 (HR = 3.22, 95% CI: 1.55–6.69, *p* = 0.022; Figure S1C) datasets. This consistent evidence across independent cohorts supports the proposition that *PIK3CA*/p110α can be a robust biomarker for adverse prognosis in breast cancer.

## 4. Discussion

In this comprehensive study, we demonstrate that elevated p110α protein expression, as detected by a standardized IHC assay in FFPE tissues, serves as a robust and independent biomarker of unfavorable prognosis in patients with stage I–III invasive breast cancer. Our principal findings are threefold. First, positive p110α expression was significantly associated with both shorter OS and RFS in our primary cohort, and it retained its independent prognostic value after adjusting for key clinicopathological variables including age, histological grade, and hormone receptor status. Second, the adverse prognostic impact of p110α was particularly pronounced in clinically relevant subgroups, including patients with early-stage (I–II) disease and those with the HR-positive/HER2-negative subtype. Third, and critically, we provided external validation at the transcriptional level, confirming that high mRNA expression of the encoding *PIK3CA* gene is consistently associated with poor survival outcomes across multiple independent, large-scale public cohorts. This concordance across different biological levels (protein and mRNA) and patient populations significantly strengthens the biological and clinical relevance of our findings.

The oncogenic role of the PI3K/AKT pathway in breast cancer is largely driven by the high frequency of *PIK3CA* mutations, one of the most common genetic aberrations in this malignancy [10,18,19]. However, the prognostic utility of these mutations has yielded conflicting results, partly due to the complex landscape of co-mutations and tumor heterogeneity [20]. Our study adds a crucial layer to this understanding by shifting the focus from the genetic alteration to the functional protein product. We establish a clear link between the abundance of the p110α protein itself and aggressive tumor behavior. This aligns with the growing recognition that the functional output of an oncogenic pathway, as reflected by protein expression or phosphorylation status, may offer more direct and integrated prognostic and predictive insights than the presence of a genetic alteration alone [21]. For instance, the activation state of the AKT/mTOR pathway, downstream of PI3K, has been shown to be a superior predictor of response to targeted therapies compared to mutational status in some contexts [22,23,24]. Our finding that p110α expression was an independent adverse prognostic factor of IBC, even after adjusting for established clinicopathological variables, underscores its potential as a complementary biomarker for risk stratification that captures the pathway’s functional activity.

A pivotal strength of our work lies in the multi-level validation strategy. Similarly to the approach taken by Sreekumar et al. [25] and other contemporary biomarker studies [26], we complemented our central IHC findings with an analysis of independent transcriptional datasets. This external validation confirmed that high *PIK3CA* mRNA expression is a harbinger of poor survival in breast cancer. The consistency between protein and mRNA levels across different technological platforms and patient cohorts strongly suggests that the observed association is not an artifact of a single cohort or detection method but represents a fundamental characteristic of a more aggressive disease subset. This dual-validation framework significantly enhances the credibility and generalizability of our conclusions, positioning *PIK3CA*/p110α dysregulation as a central and reliable determinant of tumor aggressiveness.

Our subgroup analyses yielded clinically insightful observations. Of particular note is the observation that p110α positivity was significantly associated with diminished survival among patients diagnosed with stage I–II, HR-positive/HER2-negative breast cancer. This patient population, while generally having a favorable prognosis, exhibits considerable heterogeneity in clinical outcomes, and the decision regarding the intensity and duration of adjuvant therapy can be challenging [27]. Current clinical guidelines primarily rely on clinicopathological parameters and a limited set of molecular assays for risk assessment. Our data indicate that p110α IHC could help refine this stratification by pinpointing patients within this ostensibly lower-risk group who are at a heightened risk of recurrence. This could potentially guide more intensive surveillance schedules or the consideration of adjuvant therapies targeting the PI3K pathway, especially since PI3K inhibitors are now part of the treatment arsenal for HR-positive advanced breast cancer [28]. The technical simplicity, cost-effectiveness, and widespread availability of IHC in routine pathology workflows make the assessment of p110α highly feasible for rapid clinical translation. While IHC was employed in this study to assess p110α protein expression directly within the tumor tissue architecture, alternative methods such as real-time PCR (RT-PCR) could quantify *PIK3CA* mRNA levels. Each technique offers distinct advantages: IHC provides protein-level and spatial information relevant to clinical pathology workflows, whereas RT-PCR offers high sensitivity and precise quantification. The optimal methodological choice may depend on the specific clinical or research question, and future studies could benefit from a multi-platform validation approach.

To elucidate potential mechanisms underlying the poor prognosis associated with p110α overexpression, we can infer from the existing literature. Beyond its canonical role in promoting cell proliferation and survival, p110α hyperactivation has been linked to features of tumor aggressiveness. It can induce epithelial–mesenchymal transition (EMT), a key process in metastasis, and enhance the tumor’s capacity for invasion [6,29,30]. Furthermore, dysregulated PI3K signaling has been implicated in shaping an immunosuppressive tumor microenvironment (TME) by modulating the function of tumor-infiltrating lymphocytes and myeloid cells, which could compromise anti-tumor immunity and contribute to treatment resistance [31,32,33,34]. While our study did not directly investigate the TME, the consistent poor prognosis linked to p110α across cohorts suggests its role may extend beyond cell-autonomous effects. Future studies should explore the relationship between p110α expression and the immune contexture within breast tumors.

Of course, our research has several limitations that must be acknowledged. First, its retrospective and single-center design inevitably introduces the potential for selection bias. Second, although the total sample size is acceptable, the number of cases in some molecular subgroups (such as triple-negative or HER2-positive breast cancer) was limited, constraining the statistical power to draw robust conclusions within these specific entities. Third, and most importantly, we were unable to concurrently analyze *PIK3CA* mutation status in our cohort due to constraints in archival tissue availability and dedicated funding for genomic sequencing in this retrospective setting. This precludes a direct comparison between the prognostic contributions of the genetic mutation and the protein overexpression, and leaves open the question of their potential synergy or independence. It is plausible that tumors with both a *PIK3CA* mutation and high p110α protein expression represent a distinct, hyper-activated subtype with the worst outcomes. Fourth, the IHC analysis relied on a single antibody source for detecting p110α. While this antibody demonstrated stable performance in our pilot studies, the use of an alternative antibody from a different source could further strengthen the specificity and generalizability of our findings. Furthermore, while our study focused specifically on p110α protein expression, the inclusion of other related pathway markers (e.g., phospho-AKT, *PTEN*) in the immunohistochemical analysis could have provided a more comprehensive view of PI3K/AKT pathway activation.

These limitations, however, clearly delineate the direction for future research. Our findings warrant verification in large-scale, multi-center prospective studies. Such efforts should aim to enrich data across all molecular subtypes and, most critically, integrate genomic data (*PIK3CA* mutation status) with proteomic data (p110α expression and potentially AKT phosphorylation) to fully unravel the complex regulatory mechanisms and relative clinical contributions of different levels of PI3K pathway activation. Investigating p110α’s value in predicting response to PI3K/AKT/mTOR pathway inhibitors in the adjuvant setting represents another future avenue.

## 5. Conclusions

This study provides compelling and multi-validated evidence that p110α protein expression is an independent biomarker of unfavorable prognosis in stage I–III invasive breast cancer. When combined with external mRNA-level validation, our findings position p110α as a robust and clinically relevant indicator of aggressive disease biology. The detection of p110α via IHC, a readily applicable clinical tool, may therefore improve prognostic assessment and could ultimately contribute to more personalized, risk-adapted treatment strategies for patients with breast cancer.

## Figures and Tables

**Table 1:** Clinicopathological characteristics of included IBC patients.

Characteristics	*n*	%
Age (years)		
<55	113	70.2
≥55	48	29.8
Menstrual status		
Postmenopausal	77	47.8
Premenopausal	84	52.2
Histological grade		
1–2	112	69.6
3	49	30.4
Tumor size		
≤5 cm	149	92.5
>5 cm	12	7.5
Lymph node metastasis		
No	85	52.8
Yes	76	47.2
Clinical stage		
I–II	116	72.0
III	45	28.0
HR status		
Negative	46	28.6
Positive	115	71.4
HER2 status		
Negative	109	67.7
Positive	52	32.3
p110α expression		
Negative	66	41.0
Positive	95	59.0

Abbreviations: IBC, invasive breast cancer; HR, hormone receptor; HER2, human epidermal growth factor receptor 2; *n*, number of patients; %, percentage.

**Table 2:** Association between p110α protein expression and clinicopathological characteristics in the IBC cohort.

Characteristics	*n*	p110α-Negative (*n*, %)	p110α-Positive (*n*, %)	*p*-Value
Age (years)				
<55	113	46 (40.7)	67 (59.3)	0.910
≥55	48	20 (41.7)	28 (58.3)	
Menstrual status				
Postmenopausal	77	37 (48.1)	40 (51.9)	0.081
Premenopausal	84	29 (34.5)	55 (65.5)	
Histological grade				
1–2	112	52 (46.4)	60 (53.6)	0.034
3	49	14 (28.6)	35 (71.4)	
Tumor size				
≤5 cm	149	63 (42.3)	86 (57.7)	0.242
>5 cm	12	3 (25.0)	9 (75.0)	
Lymph node metastasis				
No	85	32 (37.6)	53 (62.4)	0.361
Yes	76	34 (44.7)	42 (55.3)	
Clinical stage				
I–II	116	48 (41.4)	68 (58.6)	0.873
III	45	18 (40.0)	27 (60.0)	
HR status				
Negative	46	17 (37.0)	29 (63.0)	0.510
Positive	115	49 (42.6)	66 (57.4)	
HER2 status				
Negative	109	39 (35.8)	70 (64.2)	0.051
Positive	52	27 (51.9)	25 (48.1)	

Abbreviations: IBC, invasive breast cancer; HR, hormone receptor; HER2, human epidermal growth factor receptor 2; *n*, number of patients; %, percentage.

**Table 3:** Effect of P110α expression on OS in IBC patients by univariate and multivariate Cox regression analysis.

Characteristics	Univariate		Multivariate	
	HR (95%CI)	*p*-Value	HR (95%CI)	*p*-Value
p110α expression				
Negative	1.00 [Reference]		1.00 [Reference]	
Positive	2.30 (1.22–4.33)	0.010	2.00 (1.05–3.80)	0.034
Age (years)				
<55	1.00 [Reference]		1.00 [Reference]	
≥55	2.13 (1.13–4.01)	0.020	1.75 (0.92–3.34)	0.088
Menstrual status				
Postmenopausal	1.00 [Reference]			
Premenopausal	1.41 (0.80–2.49)	0.239		
Histological grade				
1–2	1.00 [Reference]		1.00 [Reference]	
3	2.63 (1.50–4.62)	0.001	2.24 (1.26–3.97)	0.006
Tumor size				
≤5 cm	1.00 [Reference]			
>5 cm	1.79 (0.71–4.51)	0.219		
Lymph node metastasis				
No	1.00 [Reference]			
Yes	0.84 (0.48–1.48)	0.555		
Tumor size				
I–II	1.00 [Reference]			
III	0.77 (0.40–1.48)	0.438		
HR status				
Negative	1.00 [Reference]			
Positive	1.02 (0.55–1.90)	0.942		
HER2 status				
Negative	1.00 [Reference]			
Positive	0.55 (0.28–1.08)	0.081		

Abbreviations: IBC, invasive breast cancer; HR, hormone receptor; HER2, human epidermal growth factor receptor 2; OS, overall survival; HR (in Cox models), hazard ratio; CI, confidence interval.

**Table 4:** Effect of P110 α expression on RFS in IBC patients by univariate and multivariate Cox regression analysis.

Characteristics	Univariate		Multivariate	
	HR (95%CI)	*p*-Value	HR (95%CI)	*p*-Value
p110α expression				
Negative	1.00 [Reference]		1.00 [Reference]	
Positive	1.92 (1.11–3.34)	0.020	1.92 (1.10–3.34)	0.022
Age (years)				
<55	1.00 [Reference]		1.00 [Reference]	
≥55	1.86 (1.04–3.33)	0.035	1.79 (1.07–2.99)	0.028
Menstrual status				
Postmenopausal	1.00 [Reference]		1.00 [Reference]	
Premenopausal	1.75 (1.04–2.97)	0.036	1.66 (0.99–2.81)	0.057
Histological grade				
1–2	1.00 [Reference]		1.00 [Reference]	
3	1.87 (1.12–3.11)	0.017	1.01 (0.78–1.32)	0.936
Tumor size				
≤5 cm	1.00 [Reference]			
>5 cm	1.27 (0.51–3.18)	0.607		
Lymph node metastasis				
No	1.00 [Reference]			
Yes	0.89 (0.53–1.47)	0.637		
Tumor size				
I–II	1.00 [Reference]			
III	1.01 (0.58–1.77)	0.964		
HR status				
Negative	1.00 [Reference]			
Positive	0.98 (0.56–1.71)	0.941		
HER2 status				
Negative	1.00 [Reference]			
Positive	0.77 (0.46–1.38)	0.416		

Abbreviations: IBC, invasive breast cancer; HR, hormone receptor; HER2, human epidermal growth factor receptor 2; RFS, relapse-free survival; HR (in Cox models), hazard ratio; CI, confidence interval.

## Data Availability

The data presented in this study are not publicly available due to ethical and legal restrictions related to patient confidentiality.

## Supplementary Materials

The following supporting information can be downloaded at https://www.mdpi.com/article/10.3390/cancers18020301/s1. Figure S1: External validity investigation using the open access portal PROGgeneV2. (A) In the TCGA-BRCA dataset (N = 594), high *PIK3CA* mRNA expression was associated with an increased risk of death (HR = 1.82, 95% CI: 1.30–2.53, *p* < 0.001). (B) In the GSE25055 dataset (N = 309), high *PIK3CA* mRNA expression was associated with an increased risk of relapse (HR = 1.62, 95% CI: 1.12–2.35, *p* = 0.011). (C) In the GSE10886 dataset (N = 156), high PIK3CA mRNA expression was associated with an increased risk of relapse (HR = 3.22, 95% CI: 1.55–6.69, *p* = 0.002). Table S1: REMARK Checklist for Reporting Tumor Marker Prognostic Studies; Table S2: Subgroup analysis of p110α expression and OS in IBC patients; Table S3: Subgroup analysis of p110α expression and RFS in IBC patients.
